# A Hybrid Framework for Red Blood Cell Labeling Using Elliptical Fitting, Autoencoding, and Data Augmentation

**DOI:** 10.3390/jimaging11090309

**Published:** 2025-09-09

**Authors:** Bundasak Angmanee, Surasak Wanram, Amorn Thedsakhulwong

**Affiliations:** 1 Department of Physics, Faculty of Science, Ubon Ratchathani University, Ubon Ratchathani 34190, Thailand; amorn.t@ubu.ac.th; 2 Department of Pathology, College of Medicine and Public Health, Ubon Ratchathani University, Ubon Ratchathani 34190, Thailand; mdsurawa@ubu.ac.th

**Keywords:** red blood cell morphology, autoencoder, ellipse fitting, unsupervised clustering, data augmentation, anemia, thalassemia

## Abstract

This study aimed to develop a local dataset of abnormal RBC morphology from confirmed cases of anemia and thalassemia in Thailand, providing a foundation for medical image analysis and future AI-assisted diagnostics. Blood smear samples from six hematological disorders were collected between April and May 2025, with twelve regions of interest segmented into approximately 34,000 single-cell images. To characterize cell variability, a convolutional autoencoder was applied to extract latent features, while ellipse fitting was used to quantify cell geometry. Expert hematologists validated representative clusters to ensure clinical accuracy, and data augmentation was employed to address class imbalance and expand rare morphological types. From the dataset, 14,089 high-quality single-cell images were used to classify RBC morphology into 36 clinically meaningful categories. Unlike existing datasets that rely on limited or curated samples, this dataset reflects population-specific characteristics and morphological diversity relevant to Southeast Asia. The results demonstrate the feasibility of establishing scalable and interpretable datasets that integrate computational methods with expert knowledge. The proposed dataset serves as a robust resource for advancing hematology research and contributes to bridging traditional diagnostics with AI-driven clinical support systems.

## 1. Introduction

### 1.1. Background

Red blood cell (RBC) morphology is fundamental to the diagnosis of hematological disorders, including thalassemia, iron deficiency anemia, and hemolytic diseases [1,2]. Careful assessment of cell shape, size, and staining provides critical information for identifying disease type, assessing severity, and guiding treatment. The significance of morphological examination is particularly evident in Southeast Asia, where inherited hemoglobinopathies are highly prevalent compared with Western populations. In Thailand, for example, the carrier rate of thalassemia is estimated at 30–40%, while HbE carriers exceed 50% in some regions. Severe cases are associated with mortality rates greater than 1 per 100,000 individuals annually [3,4,5,6]. These statistics highlight the disproportionate global burden of hematological disorders and underscore the urgent need for scalable and regionally adaptable diagnostic strategies.

In clinical practice, light microscopy performed by expert hematologists remains the gold standard for evaluating RBC morphology. However, this process typically requires 20–30 min per slide and is prone to inter-observer variability, with disagreement rates of 15–20% for subtle or abnormal morphologies [7]. Such limitations—time intensity, human error, and subjectivity—restrict scalability, particularly in low-resource regions where trained experts are scarce. Recent advances in computer vision and deep learning have accelerated the progress toward automated RBC classification [8,9]. Nevertheless, most existing models rely on small, curated datasets with fewer than 10,000 annotated cells, often derived from Western cohorts [10,11,12,13]. This raises concerns about population bias when applied across regions [14,15]. Moreover, curated datasets are typically acquired under controlled laboratory conditions, which fail to capture real-world challenges such as overlapping cells, heterogeneous staining, and background artifacts. This “technical reality gap” has limited the clinical translation of automated approaches.

Several computational strategies have been explored to mitigate these challenges, including whole-slide image (WSI) region-of-interest (ROI) extraction, single-cell segmentation, unsupervised clustering, shape-based modeling, expert-in-the-loop validation, and augmentation of rare classes. While these efforts demonstrate promising advances, they still face limitations in scalability, robustness, and clinical interpretability.

### 1.2. Related Works

Building upon the limitations outlined above, several computational strategies have been proposed to improve RBC image analysis. These approaches can be grouped into whole-slide processing, single-cell segmentation, unsupervised learning, shape-based modeling, expert-in-the-loop refinement, and rare-class augmentation.

WSI processing enables digitization of full peripheral smears, forming a foundation for automated morphology assessment. Yet, many studies still rely on cropped or fixed-field images collected under controlled conditions, which fail to capture real-world variability such as overlaps, heterogeneous staining, and artifacts [2,16]. The absence of standardized ROI protocols further restricts reproducibility, while heuristic or random sampling lacks adaptive mechanisms for density, aggregation, or diagnostic saliency. Thus, WSI-based ROI analysis remains underexplored but critical for large-scale annotation frameworks [17]. Reliable RBC separation remains a major challenge in computational hematology. Classical methods such as thresholding, edge detection, and watershed are computationally efficient but consistently fail with overlapping or touching cells, producing artifacts that compromise morphological accuracy [18]. Deep learning approaches, including U-Net and Mask R-CNN, improve segmentation but require costly pixel-level annotations and often generalize poorly to noisy smears with heterogeneous staining and dense clusters. The absence of standardized ROI sizing further introduces dataset inconsistencies, complicating downstream classification and limiting reproducibility [19]. In response to the high cost of manual annotation, unsupervised learning has emerged as a promising strategy for RBC analysis, reducing reliance on costly manual annotation [20]. Autoencoders can capture latent morphological representations without explicit labels, and these features can be clustered using algorithms such as k-means or DBSCAN to group morphologically similar cells, enabling scalable pseudo-label generation. However, most studies in this area focus on WBCs, leaving abnormal RBC morphologies underexplored despite their clinical relevance. In addition, systematic comparisons between encoder backbones—such as dense autoencoders versus convolutional neural networks—remain limited, even though architectural choices may significantly affect the capture of subtle, shape-dependent features under variable imaging conditions [21]. Parallel to representation learning, shape-based modeling has long been applied in hematological image analysis to quantify cellular morphology using geometric descriptors such as area, perimeter, circularity, and eccentricity [22]. These handcrafted features are simple and interpretable but lose effectiveness in real-world smears where cell boundaries are distorted by overlaps or staining artifacts. Ellipse fitting has been proposed as a more robust alternative, as healthy RBCs are approximately elliptical. This method systematically quantifies elongation and circularity, bridging traditional morphology-based assessments with computational pipelines [23,24]. Despite its potential, ellipse fitting remains underutilized in modern deep learning frameworks, where it could provide a valuable geometric prior to enhance interpretability and classification [25]. HITL strategies balance automation with expert oversight, allowing hematologists to refine or validate AI outputs instead of annotating thousands of cells manually, thereby reducing labeling effort while preserving diagnostic accuracy [26]. This approach has demonstrated effectiveness in domains such as histopathology and radiology, where interactive feedback cycles enhance model performance and user trust [27,28]. Despite successes elsewhere, HITL adoption in RBC morphology is limited. Most studies emphasize supervised classification or post hoc validation, lacking expert input during clustering or pseudo-labeling. Few systems offer intuitive interfaces for correction, leaving HITL underutilized in hematology yet presenting clear opportunities for integration [29]. Finally, severe class imbalance remains one of the most persistent challenges in RBC datasets. Rare morphologies such as teardrop cells, fragmented cells, and target cells are clinically significant indicators of hematological disorders, yet they occur infrequently, biasing models toward majority classes and reducing sensitivity to these critical phenotypes [30]. Classical balancing strategies such as oversampling and SMOTE have been attempted, but they often generate redundant or unrealistic samples that compromise morphological authenticity and limit clinical applicability [31]. More recently, generative models such as GANs have been applied to create synthetic cell images for minority classes [32]. However, many ignore shape constraints, producing unrealistic samples. Geometry-aware methods, including ellipse-based augmentation, offer deformable yet biologically plausible transformations that preserve morphology while increasing diversity. Still, these approaches remain underexplored and lack large-scale validation in hematology [33,34].

A concise comparison of representative methods is summarized in Table 1, highlighting their principal advantages and limitations. Collectively, prior studies demonstrate substantial progress, yet most continue to address these challenges in isolation. Only a limited number of works attempt to integrate WSI processing, unsupervised clustering, geometric priors, expert-in-the-loop validation, and rare-class augmentation into a unified framework, underscoring the need for a holistic and clinically relevant approach to abnormal RBC annotation.

### 1.3. Research Gap and Aim

Prior research has advanced RBC image analysis from whole-slide preprocessing to single-cell classification. However, as summarized in Table 1, most methods have been developed in isolation rather than as part of a unified workflow for real-world smears. Key limitations persist as follows: (i) limited adoption of unsupervised learning for abnormal RBC morphology, especially in non-Western cohorts; (ii) scarce integration of geometric priors such as ellipse fitting into modern frameworks; (iii) minimal application of expert-in-the-loop strategies for clustering and pseudo-label validation; and (iv) inadequate augmentation of rare morphologies, which restricts balanced dataset development. To overcome these shortcomings, this study developed an end-to-end hybrid framework that integrates shape-based segmentation, latent space clustering, expert-guided pseudo-label refinement, and deformable augmentation. Unlike previous works relying on curated Western datasets, the proposed approach was tailored to peripheral smears from Thai patients with anemia and thalassemia. The overarching aim was to generate scalable, interpretable datasets that connect computational modeling with clinical diagnostic utility.

## 2. Materials and Methods

**Figure 1:**
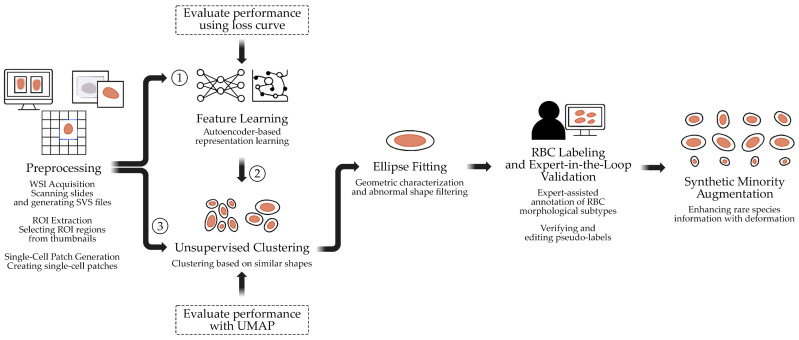
Semi-automated pipeline for RBC annotation. The process integrates preprocessing, feature learning, clustering, ellipse fitting, expert validation, and minority augmentation. The circle numbers (1, 2, 3) denote the sequential order of data flow and experimental steps in the pipeline.

The proposed workflow integrates WSI processing, unsupervised representation learning, shape-based priors, and expert-in-the-loop validation to address challenges of manual annotation and data imbalance in hematological image analysis. As illustrated in Figure 1, the pipeline begins with the acquisition of high-resolution WSIs from clinically confirmed cases (Section 2.1) and extraction of diagnostically relevant ROIs (Section 2.2). Uniform single-cell patches are generated via grid sampling and filtering (Section 2.3). Latent morphological features are then learned using autoencoder-based representation learning (Section 2.4) and clustered by unsupervised methods (Section 2.5). Ellipse fitting provides geometric characterization and filters abnormal shapes (Section 2.6). Cluster results are refined through expert-in-the-loop validation (Section 2.7), where specialists confirm or adjust pseudo-labels. Finally, synthetic minority augmentation based on deformable ellipse transformations is applied to mitigate class imbalance among rare morphologies (Section 2.8).

### 2.1. Dataset Collection and Image Acquisition

**Figure 2:**
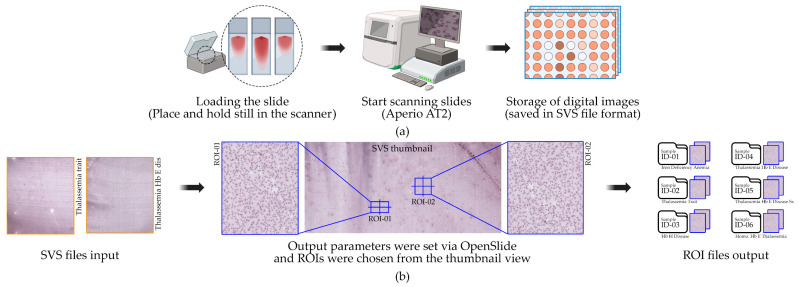
Workflow of slide scanning and ROI selection using OpenSlide: (**a**) Peripheral blood smear slides were scanned using Aperio AT2, generating high-resolution SVS files. (**b**) ROIs were defined via OpenSlide from thumbnail views, with each ROI exported as an independent dataset for subsequent RBC analysis.

Six WSIs of peripheral blood smears were collected from Thai patients with clinically confirmed hematological diagnoses, including iron deficiency anemia (IDA), thalassemia trait (TT), Hb H disease (HbH), Hb E/β-thalassemia (HbE/β-thal), severe Hb E/β-thalassemia (HbE/β-thal Sx), and homozygous Hb E thalassemia (Homo HbE). The diagnostic spectrum is summarized in Table 2. All cases were confirmed by hematologists based on standard laboratory tests and microscopic examination prior to slide preparation. No demographic or personal identifiers were collected, in accordance with de-identification and privacy protection guidelines. Peripheral blood smears were prepared using routine hematology protocols and stained with Wright-Giemsa to enhance red blood cell morphology visualization [35]. The slides were digitized using a whole-slide imaging (WSI) scanner at 40× magnification, producing high-resolution SVS-format digital slides with a scanning resolution of 0.1658 µm/pixel, as illustrated in Figure 2a [36,37]. Each WSI covered the entire smear area and served as the primary source for subsequent ROI extraction, single-cell segmentation, and morphological analysis. This study was approved by the Institutional Review Board of Ubon Ratchathani University, approval number UBU–REC–77/2568, and conducted in accordance with the Declaration of Helsinki. The dataset is not publicly available due to patient confidentiality; however, anonymized images can be accessed upon reasonable request to the corresponding author, subject to institutional ethics approval.

### 2.2. ROI Selection from WSI

From each WSI, two diagnostically informative regions of interest (ROIs) were extracted, resulting in 12 ROIs across the six hematological cases. To minimize subjectivity and ensure reproducibility, quantitative screening criteria were defined prior to selection. Candidate ROIs were required to satisfy the following conditions: (i) dimensions between 2000 and 8000 pixels in width, guaranteeing sufficient coverage for representative single-cell extraction; (ii) proportion of staining artifacts, debris, or scanner-induced noise not exceeding 5–10% of the total ROI area; and (iii) clumping filter, whereby overlapping or aggregated red blood cells occupied no more than 15% of the region [37]. These thresholds were chosen to balance diagnostic clarity with data cleanliness, thereby excluding regions that were either too sparse, overly dense, or compromised by preparation artifacts, as illustrated in Figure 2b. After preliminary screening, ROI candidates were independently reviewed by two hematologists with over 10 years of diagnostic experience. Reviewers were blinded to downstream analyses and asked to confirm whether candidate ROIs represented morphologically informative areas consistent with the patient’s diagnosis. To further minimize selection bias, only ROIs where both experts reached a consensus were retained, yielding two final ROIs per diagnostic category. All ROIs were extracted at native scanning resolution using the OpenSlide library [38], ensuring precise coordinate mapping and reproducible cropping from SVS files. The images were saved in lossless PNG format, preserving diagnostic quality for subsequent segmentation and morphological analysis. Further details are provided in Appendix A.

### 2.3. Single-Cell Patch Extraction

**Figure 3:**
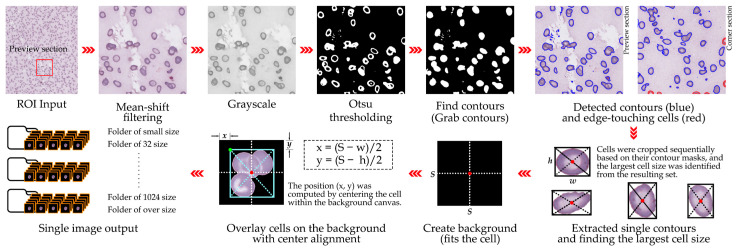
Processing pipeline for single-cell patch extraction from ROIs. The red square box highlights the selected region enlarged for preview. The workflow including segmentation, watershed-based separation, bounding box generation, artifact filtering, cell centering, and final patch export.

A total of 12 ROIs selected from WSIs were processed to extract single-cell patches of RBCs for downstream analysis. Cell detection was performed using a segmentation-based approach incorporating global thresholding, contour detection, and morphological operations. Segmentation masks were generated and refined using the watershed algorithm [39] to separate individual cells from touching clusters. For each segmented cell, a bounding box was derived from the binary mask and cropped to create a single-cell patch. To ensure accurate identification of isolated cells versus touching cells, a maximum local peak detection method was applied to centroid distributions within clusters [40]. Validated single-cell patches were overlaid on a clean background and centered to standardize positioning. Patches were saved in PNG format with a structured directory system separating: (i) single isolated cells, (ii) overlapping cells, (iii) broken or artifact cells, (iv) background or staining artifacts, (v) small particles, and (vi) cells touching image edges. This systematic organization facilitated both quality control and downstream dataset preparation [2]. The patch size for this study was set at 128 × 128 pixels, optimized for our ROI resolution (0.1658 µm/pixel) and compatibility with subsequent deep learning models. In addition, these dimensions ensured that the cellular details such as membrane irregularities, central pallor, and morphological variations in abnormal RBCs were adequately preserved while still being computationally manageable for large-scale training. For other contexts, patch sizes can be adapted (e.g., 32, 128, or 256 pixels) depending on the desired field of view and computational constraints [41]. Filtering criteria were strictly applied to exclude: (i) overlapping cells, (ii) fragmented cells or artifacts, (iii) background regions or staining debris, (iv) small non-cellular particles, and (v) cells truncated at image edges. Such stringent exclusion criteria minimized noise and prevented misleading inputs that could negatively impact the learning process of convolutional neural networks. Furthermore, retained cells underwent manual inspection in a subset of ROIs to confirm segmentation accuracy, thereby reinforcing dataset reliability. The detailed cell extraction process is visualized in Figure 3, while Algorithm 1 formally outlines the procedural steps, including segmentation, bounding box generation, artifact filtering, and patch centering.
**Algorithm 1.** Pseudocode of the RBC single-cell extraction and resizing techniqueRBC_Extraction(image_path, output_dir)  load image from image_path  apply mean-shift filtering to image  → shifted  convert shifted image to grayscale  → gray  apply Otsu thresholding to gray   → thresh  find contours from thresh      → cnts  **for** each contour c **in** cnts **do**    crop image and mask around contour → image_crop, mask_crop    **if** mask_crop is valid then      check if cell touches border:        **if** true:          save touching cell to “touching” folder        **else**:           extract RBC from mask          determine RBC size:          **if** size ≤ 16 px: overlay to 32 × 32 and save as “small”          **else if** size ≤ 32 px: overlay to 32 × 32 and save as “32 size”          **else if** size ≤ 128 px: overlay to 128 × 128 and save as “128 size”          **else if** size ≤ 256 px: overlay to 256 × 256 and save as “256 size”          **else if** size ≤ 512 px: overlay to 512 × 512 and save as “512 size”          **else if** size ≤ 1024 px: overlay to 1024 × 1024 and save as “1024 size”          **else**: save as “oversize”    **else**:  **end for**  **save** processing results (original, filtered, gray, mask, contours)  **return** RBC_dataset_summary

The extracted patches were systematically organized into two datasets to facilitate reproducible analysis and structured evaluation. Dataset 1 comprised only isolated single-cell images, including RBCs, WBCs, and PLTs. All patches in this dataset were resized to 128 × 128 pixels, corresponding to the scanning resolution and optimized for compatibility with deep learning models. Dataset 1 thus served as the primary source of training and evaluation data for downstream classification tasks, ensuring that only diagnostically relevant and clearly separated cells were included.

**Figure 4:**
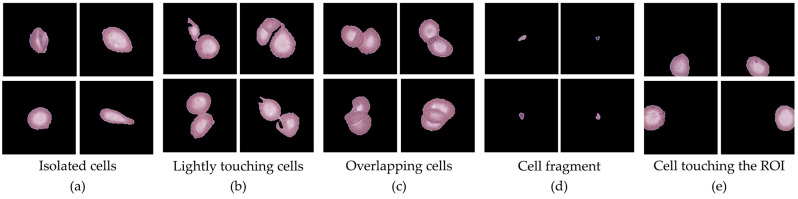
Representative examples of cell categories identified during preprocessing are as follows: (**a**) isolated single cells retained for classification; (**b**) small separable clusters excluded to avoid ambiguity; (**c**) larger overlapping clusters with indistinguishable cells; (**d**) fragments or platelets, with only platelets retained; and (**e**) cells truncated at ROI edges, excluded from analysis.

In contrast, Dataset 2 contained additional categories identified during preprocessing that did not meet the strict inclusion criteria for Dataset 1. These categories included: (i) small clusters of two or three cells that could be morphologically separated but were excluded to avoid ambiguity, (ii) larger overlapping clusters where individual cells could not be distinctly resolved, (iii) fragmented or artifact-containing cells, (iv) background regions or staining debris, (v) small non-cellular particles, and (vi) cells truncated by ROI boundaries. While Dataset 2 was excluded from model training, it was retained as a reference set for quality control, error analysis, and documentation of the distribution of artifacts encountered in clinical smear imaging. Representative examples of these categories are provided in Figure 4, and detailed image counts across hematological conditions are summarized in Table 3 and Table 4.

### 2.4. Latent Feature Learning Using Autoencoders

**Figure 5:**
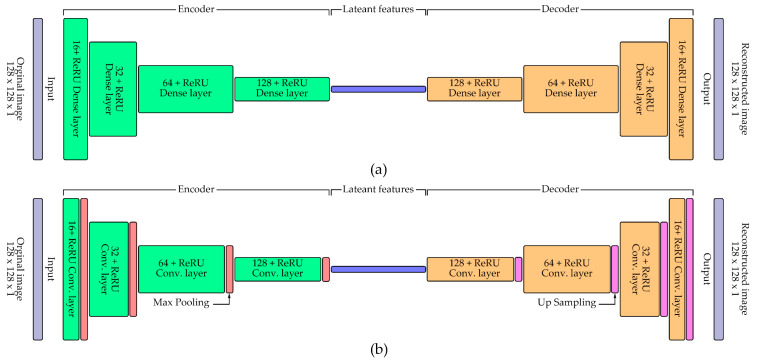
Architectures of the autoencoder models used for RBC feature extraction: (**a**) Dense autoencoder consisting of fully connected layers for encoding and decoding single-cell patches; (**b**) Convolutional autoencoder employing convolution, max pooling, and upsampling layers to capture spatial features and reconstruct RBC images.

Latent morphological features of RBC single-cell patches were extracted using two encoder designs: a dense autoencoder and a CNN-based autoencoder. Both were implemented in TensorFlow/Keras [42], with the architecture shown in Figure 5. Input patches (128 × 128 pixels, grayscale) were normalized to [0, 1]. The dense autoencoder included a flattened input, a 64-unit latent layer with ReLU, and decoding layers with sigmoid activation, reshaping to the original size. The CNN autoencoder applied convolution and max-pooling layers for encoding, mirrored by upsampling and convolutional layers for reconstruction. Training used cross-validation with an 80/20 train–validation split per fold. Models were trained for 200 epochs, batch size 64, on an NVIDIA GeForce RTX 1650 GPU. Binary cross-entropy served as the loss, and performance was tracked using reconstruction loss [43]. Training histories were saved in CSV format, while loss curves supported convergence assessment. Encoders from both models were later extracted for downstream unsupervised clustering.

### 2.5. Unsupervised Clustering

The best-performing encoder selected from the autoencoder experiments was utilized to extract latent features of single-cell RBC images for clustering. These latent feature vectors were subsequently grouped using k-means clustering, implemented via the scikit-learn library [44]. The optimal number of clusters (k) was determined experimentally by iterative testing and qualitative expert evaluation, as no fixed ground truth labels were available. The quality of clustering was assessed primarily through expert visual review, wherein representative cell images from each cluster were inspected by a hematology specialist to verify morphological coherence within clusters [27]. To aid interpretation, the high-dimensional latent space was reduced to two dimensions using Uniform UMAP [45], enabling visualization of cluster separability and distribution patterns. The clustering process was conducted using TensorFlow and scikit-learn. Resulting clusters were saved as image directories grouped by cluster ID to facilitate manual review and downstream analysis. Representative examples of clustered images were visualized to demonstrate intra-cluster similarity and inter-cluster distinctiveness (Algorithm 2).
**Algorithm 2.** Unsupervised clustering of RBC latent features using k-meansRBC_Clustering_Encoder(samples, encoder_models, num_clusters_list)  **for** each sample_id in samples **do**    initialize paths for model, images, and outputs    create output folders if not exist    load pre-trained encoder model for current sample_id    load and preprocess RBC images   → x_test    normalize pixel values (0–1)    encode images using encoder     → encoded_imgs    remove existing clustering score CSV if exists    **for** each num_clusters in num_clusters_list **do**      apply KMeans clustering (num_clusters)      compute clustering labels     → labels      calculate Silhouette Score     → sil_score      calculate Davies–Bouldin Index  → dbi_score      **save** scores to CSV log      create cluster folders and copy images based on labels      **plot** silhouette visualization per cluster      **plot** metrics comparison (Silhouette vs. DBI)      **save** plots      apply UMAP to reduce encoded_imgs to 2D      plot and save UMAP scatter plot with cluster coloring      **save** plots    **end for**    compile per-sample clustering report summarizing metrics and plots    append results to global clustering summary (multi-sample CSV)  **end for**  compute total execution time and display summary  **return** clustering_results_summary

### 2.6. Morphological Prior via Ellipse Fitting

Ellipse fitting was applied to accurately quantify RBC morphology and provide shape-based priors for classification. The primary objectives were to (i) measure cell size precisely and (ii) evaluate shape characteristics such as circularity, ellipticity, and completeness, which are essential for distinguishing subtle morphological variations [46]. Ellipse fitting was implemented using edge-based contour fitting via the cv2.fitEllipse () function in OpenCV [47]. For each segmented cell, an ellipse was fitted to the contour, from which key morphological parameters were derived, including the major axis length ($L_{major}$), minor axis length ($L_{minor}$), aspect ratio (*AR*), and ellipse-to-cell area ratio (*ER*). These were calculated as follows:

(1) $$L_{major}\;=\;max\left(a,\;b\right)$$

(2) $$L_{minor}=min\left(a,\;b\right)$$

(3) $$AR=\frac{L_{major}}{L_{minor}}$$

(4) $$ER=\frac{Cell\;area\;}{Major\;circle\;area}=\frac{Cell\;area}{\pi \;\cdot {\;\left(\frac{L_{major}}{2}\right)}^{2}}$$

Cells with *AR* ≈ 1 were considered circular, whereas higher *AR* values indicated elongation. The classify cell function (see Algorithm 3) applied threshold-based rules using *AR*, major axis length (µm), and *ER* to categorize cells into predefined groups (e.g., circular, oval, elongated) and filter out artifacts. Subsequently, morphological metrics were statistically analyzed, including *AR* for circularity, major axis length for RBC size standardization (6–8 µm), and *ER* to assess structural completeness [35]. This ensured that only morphologically valid cells were retained for downstream tasks. The full processing workflow, including contour detection, ellipse fitting, feature computation, and classification, is summarized in Algorithm 4, which automated morphological quantification while maintaining interpretability. This integration of geometry-based priors improved the reliability of subsequent clustering and annotation steps by removing irregular cells and enhancing feature quality.

In practice, the *AR* thresholds were applied according to the rules defined in Algorithm 3. Cells with *AR* within ±5% of unity were assigned to the circular group, as this range corresponds to morphologically normal red blood cells that typically appear round in peripheral smears. Cells with *AR* between 5 and 60% elongation were classified as oval, with finer subdivisions at ±10%, ±20%, ±30%, ±40%, and ±60% to facilitate downstream discrimination and to capture intermediate shapes such as slightly oval versus moderately elongated cells. Cells with *AR* between 60 and 80% elongation were considered highly elongated (pencil-shaped) and were separated from oval categories to better reflect clinically abnormal morphologies such as elliptocytes or sickle-like forms. In addition to *AR*, the maximum diameter ($L_{major}$) was used to screen abnormal sizes: $L_{major}$ < 6 μm indicated microcytic RBCs, 6–8 μm represented normocytic RBCs, and $L_{major}$ > 8 μm denoted macrocytic RBCs, which aligns with hematological standards for RBC sizing. Finally, *ER* was used as a complementary filter to reduce false *AR* values caused by incomplete contours, overlapping cells, or fragmented boundaries. Unlike *AR* and size thresholds, *ER* does not follow a universal theoretical cut-off but was empirically determined as a dataset-specific quality control parameter. This parameter was optimized through expert inspection of 300 sampled cells, where *ER* > 0.50 reliably excluded artifacts while retaining morphologically valid single cells, thereby improving the robustness of the overall morphological screening pipeline.
**Algorithm 3.** RBC Morphological classification based on geometric featuresclassify_cell(ratio, length, area)  **if** ratio ≤ 1.05:     r_group = “Circle 095/”  **else if** ratio ≤ 1.10:  r_group = “Circle 090/”  **else if** ratio ≤ 1.20:  r_group = “Circle 080/”  **else if** ratio ≤ 1.40:  r_group = “Oval 060/”  **else if** ratio ≤ 1.60:  r_group = “Oval 040/”  **else**:         r_group = “Pencil/”   **if** length < 6.0:    l_group = “Micro/”  **else if** length ≤ 8.0:  l_group = “Normal/”  **else**:        l_group = “Macro/”   **if** area ≤ 0.80:     a_group = “Area 080/”  **else if** area ≤ 0.90:   a_group = “Area 090/”  **else if** area ≤ 0.95:   a_group = “Area 095/”  **else**:        a_group = “Area 100/”   **return** concatenation(l_group, r_group, a_group)

**Algorithm 4.** Ellipse-based RBC Morphology classification and clusteringRBC_Ellipse_Fitting_Clustering(data_list, image_path) **  for** each folder in data_list **do**    define folder_path    **if** folder_path exists then      **for** each image_file in folder_path **do**        load image          → image_input        convert image to grayscale   → gray        apply Otsu thresholding    → binary        find contours from binary   → contours        **for** each contour cnt in contours **do**          **if** contour length ≥ 5 then            fit ellipse to contour  → ellipse            extract ellipse parameters: center, major_ax, minor_ax, angle            compute major/minor axis lines and endpoints            convert axis lengths to micrometers (µm)            determine aspect ratio (*AR*)            generate contour and ellipse masks            compute overlap region (intersection) → inter_contours            calculate area ratio (*ER*)            annotate image with ellipse, axes, ratio, and area metrics            classify cell morphology using classify_cell() function            define output directories based on classification            save annotated and raw images into their respective folders            log extracted metrics for statistical analysis            append classification results to CSV for later clustering review      **end for**    **else**: print warning (folder not found)  **end for** export full metrics dataset and classification summary  **return** ellipse_classification_results 

### 2.7. Expert-in-the-Loop Validation

To ensure the reliability of clustering outcomes and establish clinically meaningful labels, a HITL validation strategy was implemented. RBC images were first grouped by unsupervised clustering, which produced clusters of cells with similar latent morphological features. These clusters, rather than individual cells, were then submitted to expert review, where domain specialists inspected representative images and assigned appropriate morphology labels. Two experts contributed to the review of the clusters: (i) an associate professor of Hematology specializing in physician training, and (ii) a lecturer in Biomedical Physics with expertise in image analysis. Their complementary backgrounds ensured that both clinical relevance and computational rigor guided the labeling process. The experts applied explicit criteria: (i) evaluating whether the visual coherence of each cluster corresponded to a valid RBC morphology, (ii) assigning or refining morphology labels for downstream use, and (iii) flagging clusters that contained mixed or artifact samples. Representative cells from all clusters were inspected, and a distribution chart was generated to illustrate how expert-confirmed morphologies mapped onto the unsupervised clusters. Validation was performed in two refinement cycles, during which ambiguous clusters were re-examined and noisy samples were removed. This iterative process improved label precision, minimized bias, and established confidence in the pseudo-labels. By integrating algorithmic grouping with expert oversight, the hybrid validation bridged automated clustering with clinical standards, yielding reliable annotations for robust deep learning [48,49].

### 2.8. Synthetic Minority Augmentation

To address the issue of class imbalance, data augmentation was applied to increase the representation of rare morphological subtypes of RBCs. Imbalanced datasets are a well-known challenge in medical imaging, as they often bias model training towards majority classes and degrade performance in clinically important but underrepresented categories [30]. Synthetic samples were generated using controlled geometric transformations to preserve biological plausibility. Specifically, transformations were limited to rotation, flipping, and scaling down, ensuring that augmented data remained consistent with the original morphology and did not introduce unrealistic variations [50]. For each rare class, synthetic samples were generated at three scales: 1000, 2000, and 4000 images per class, resulting in a more balanced training distribution. Augmentation operations were implemented in Python v.3.9.4 using OpenCV v.4.7.0.72, NumPy v.1.23.5, and SciPy v. 1.9.1 libraries [47]. The workflow is described in Algorithm 5, which details the sequential application of resizing, rotation, and flipping, followed by dataset reorganization and saving augmented images. The effect of augmentation was evaluated by comparing model performance before and after augmentation, consistent with prior studies demonstrating that augmentation significantly improves classification accuracy in hematological imaging tasks [24,51]. These studies showed that class-balancing augmentation enhances sensitivity to rare morphological types and stabilizes learning curves, leading to improved generalization.
**Algorithm 5.** Automated data augmentation and centering for RBC image datasetAuto_Data_Augmentation(data_list, image_path)  **for** each folder in data_list **do**    define folder_path    **if** folder_path exists then      **for** each image_file in folder_path **do**        load image        **for** each scale_factor in [0.98, 0.99, 1.00, 1.01] **do**          resize image while embedding onto black background          save augmented image          **for** each rotation angle based on num_rotations **do**            rotate resized image            **for** each flip_code in [0, 1, −1] **do**              flip rotated image (vertical, horizontal, both)    **else**: Print warning (folder not found) **  end for**  **for** each folder in data_list **do**    define folder_aug    **for** each image_file in folder_aug **do**      load augmented image   → image      apply mean-shift filtering  → shifted      convert to grayscale and apply Otsu thresholding → thresh      detect contours → cnts      **for** each contour c in cnts **do**        extract ROI with small padding        generate binary mask and apply bitwise extraction        **if** extracted cell size < 128×128:          embed cell into black 128×128 background, centered          **save** centered image  **end for** generate augmentation report summarizing transformations applied  **return** augmented_dataset_summary 

## 3. Results

### 3.1. Preprocessing Results

From the 12 ROIs, a total of 34,282 valid patches were generated across two datasets (Table 3 and Table 4). The majority of patches were single isolated cells, representing 55–70% of all images. Extracted clusters contributed 10–20%, while overlapping cells comprised 8–15%. Small fragments and edge-touching cells together accounted for less than 10% of the data. Non-cellular contaminants were minimal and did not exceed 0.5% in any ROI. Overall, the preprocessing stage produced datasets dominated by diagnostically relevant single-cell images, with artifacts and ambiguous regions remaining only as minor proportions.

### 3.2. Unsupervised Clustering Outcomes

**Figure 6:**
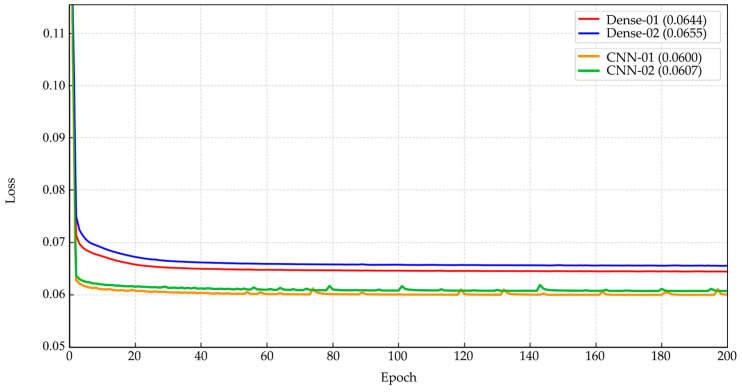
Training loss curves for dense (Dense-01, Dense-02) and CNN (CNN-01, CNN-02) models over 200 epochs. Both model types showed stable convergence, with CNN variants reaching lower final loss values (~0.0600–0.0607) compared with Dense models (~0.0644–0.0655) across both datasets.

After expert-guided filtering of single-cell patches, Dataset 1 contained 14,089 valid images, while Dataset 2 contained 11,496 images. These datasets were subsequently used to train dense autoencoder and CNN autoencoder models for unsupervised representation learning. The dense autoencoder was trained on both datasets for 200 epochs, requiring approximately 1–1.5 h per run. The minimum reconstruction losses achieved were 6.44% for Dataset 1 and 6.55% for Dataset 2. The training curves were smooth, with gradual convergence toward stable values, as illustrated in Figure 6 (top). In contrast, the CNN autoencoder required longer training times of about 7–8 h for 200 epochs, but it produced lower minimum reconstruction losses of 6.00% for Dataset 1 and 6.07% for Dataset 2. The corresponding training curves showed mild fluctuations before stabilizing at lower values than the dense autoencoder, as presented in Figure 6 (bottom). Across both datasets, reconstruction loss values ranged consistently between 6.00% and 6.55%. Figure 6 provides a comparative visualization of the convergence behaviors of the two models, highlighting differences in efficiency and reconstruction accuracy.

**Figure 7:**
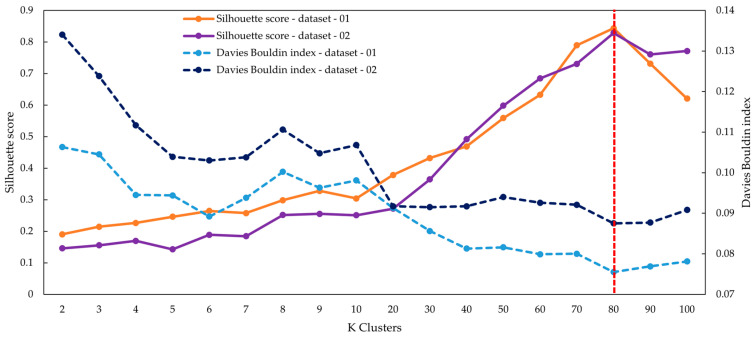
Silhouette and Davies–Bouldin indices for clustering evaluation across Dataset 1 and Dataset 2, showing more cohesive and better-separated clusters. Both datasets achieved optimal grouping near K = 80 (red dashed line).

Following feature extraction using the CNN autoencoder trained on Dataset 1 and Dataset 2, k-means clustering was systematically evaluated across a broad range of cluster sizes, from k = 2 to k = 100. As illustrated in Figure 7, both evaluation metrics showed consistent trends. The Silhouette scores increased steadily as the number of clusters rose, while the Davies–Bouldin indices decreased correspondingly. These numerical patterns indicated that clustering quality improved with higher k values, providing stronger cohesion within clusters and clearer separation between them. A closer examination of specific configurations highlighted the differences in clustering outcomes. At k = 60, clusters were relatively broad and heterogeneous, often grouping cells of different shapes, including round, oval, and elongated forms. This configuration produced notable overlaps across categories, limiting morphological resolution. At k = 70, cluster separation improved, and the overall distribution was more balanced, although several clusters still included mixed morphologies. At k = 80, clustering achieved the clearest separation and most balanced partitioning. Dataset 1 recorded a Silhouette score of 0.8432 with a Davies–Bouldin index of 0.0755, while Dataset 2 achieved a Silhouette score of 0.8287 and a Davies–Bouldin index of 0.0875. These values represented the most compact and clearly separated groups across the tested range. When the cluster size was increased to k = 90, finer morphological details began to emerge. Elongated, irregular, and fragmented cells were separated more distinctly than in previous settings. However, some groups that were stable at lower k values became divided into smaller subsets, leading to additional complexity in the cluster structure. At k = 100, the clustering process produced numerous very small clusters, which reflected subtle differences but fragmented previously coherent groups into multiple subdivisions. This high degree of fragmentation reduced the practical usefulness of the configuration, as the number of clusters substantially exceeded the scale required for straightforward interpretation. Figure 7 summarizes the behavior of clustering across both datasets, presenting quantitative changes in both Silhouette scores and Davies–Bouldin indices. The figure demonstrates that Silhouette values increased systematically while Davies–Bouldin indices decreased as k increased, and it highlights comparative outcomes at k = 60, 70, 80, 90, and 100. Together, these results illustrate the detailed clustering performance patterns observed across the experimental evaluation of different cluster configurations.

**Figure 8:**
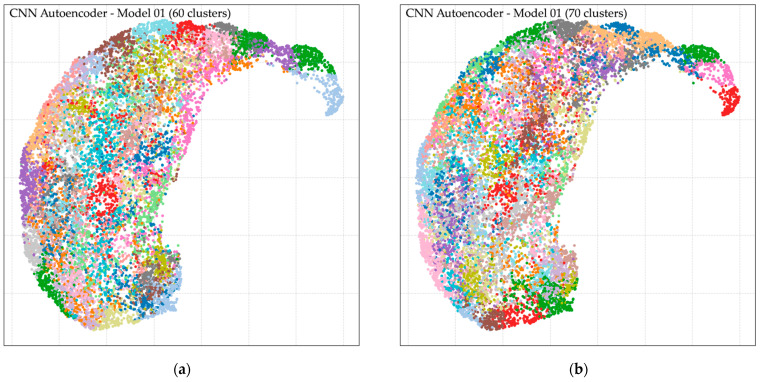

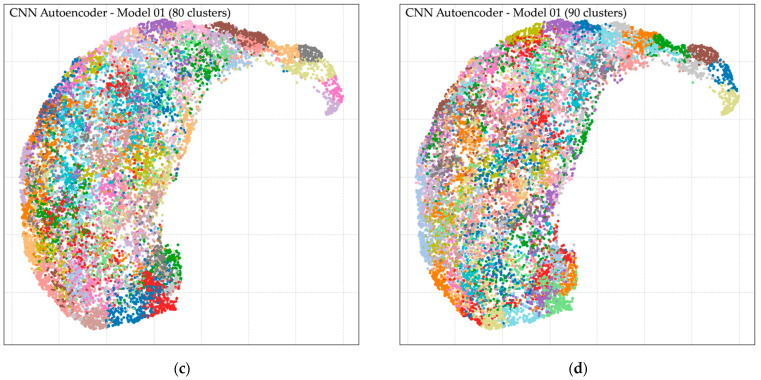
UMAP results of RBC clustering at (**a**) 60 clusters, (**b**) 70 clusters, (**c**) 80 clusters, and (**d**) 90 clusters. Colors indicate different clusters, with some colors reused due to >60 groups.

The performance of UMAP-based clustering was evaluated under multiple cluster configurations, with Figure 8 showing visualizations for 60, 70, 80, and 90 clusters. At k = 60, clusters appeared broad and heterogeneous, with several categories overlapping. At k = 70, group separation improved, but some clusters still contained ambiguous regions requiring further resolution. At k = 80, clustering achieved the clearest partitioning, producing well-defined groups with balanced granularity and reduced overlap across categories. Dataset 1 and Dataset 2 both exhibited clear separation at this configuration, with visual clusters showing minimal mixing between different morphologies. At k = 90, clustering revealed finer details, capturing elongated and irregular cells more distinctly, but the number of clusters increased and redundancy became more evident. Overall, the UMAP projections demonstrated that k = 80 produced the most stable and interpretable cluster distribution across both datasets, as visualized in Figure 8.

### 3.3. Ellipse Fitting and Expert-Guided Labeling

**Figure 9:**
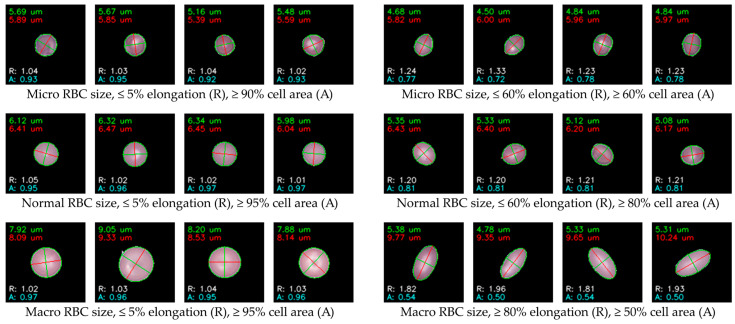
Representative results of ellipse fitting for RBCs across clusters, showing annotated values of aspect ratio (R) and ellipse-to-boundary ratio (A). The examples highlight quantitative variation in cell size, circularity, and elongation as obtained from the fitting procedure.

Ellipse fitting was applied to clustered cells to quantify RBC geometry, producing standardized measures of size, elongation ratio (R), and ellipse-to-boundary ratio (A). These descriptors enabled consistent assessment of morphological variation across datasets. The fitted ellipses provided reliable estimates of cell dimensions, distinguishing microcytic cells (<6.00 µm), normocytic cells (6.00–8.00 µm), and macrocytic cells (>8.00 µm). Elongation ratios further separated round cells from moderately elongated oval cells and highly elongated pencil-shaped cells. The ellipse-to-boundary ratio additionally differentiated well-fitted cells from incomplete or irregular boundaries, ensuring that only high-quality representations were retained. Representative outcomes of this stage are shown in Figure 9, where annotated examples illustrate categories based on size, elongation, and fitted area. The figure demonstrates that the algorithm generated clearly distinguishable cell groups, providing systematically defined morphological examples for subsequent expert validation and classification. These results indicate that ellipse fitting produced coherent geometric descriptions across clusters.

**Figure 10:**
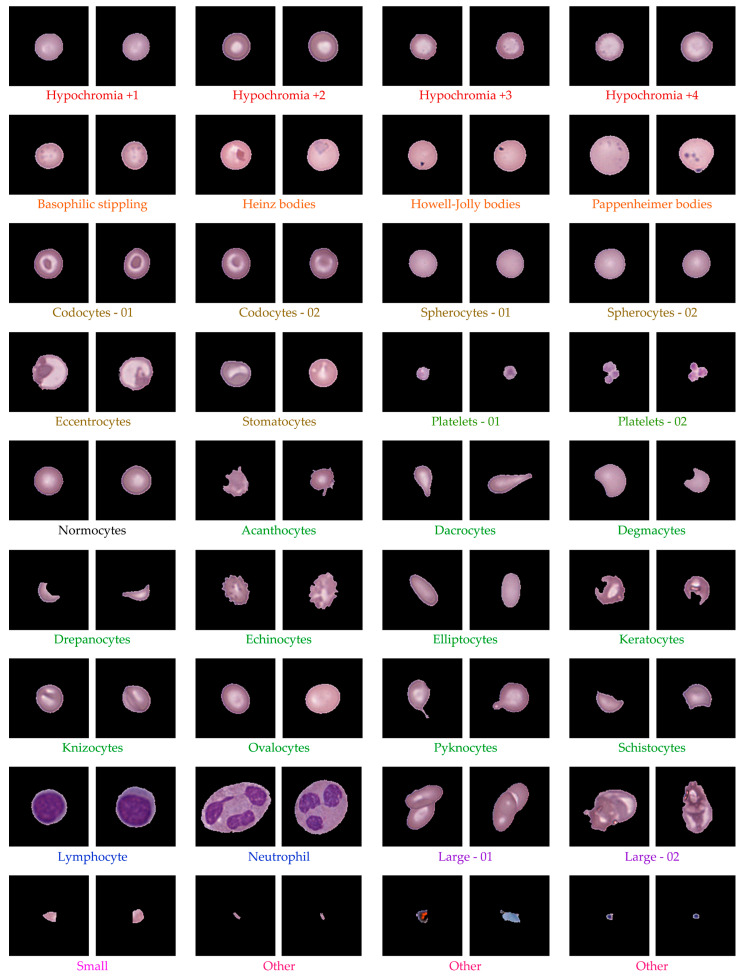
Representative RBC morphologies validated by expert review.

After ellipse fitting, the clustered data were organized into 80 groups and subsequently classified into established RBC morphologies using hematology references [52,53,54]. Two hematology experts independently reviewed all clusters, verified morphological consistency, and reassigned labels when necessary. The validation process produced a reliable classification covering a wide spectrum of morphologies. Table 5 summarizes the distribution of 14,089 single-cell images across categories including normocytes, hypochromic cells, codocytes, spherocytes, dacrocytes, elliptocytes, drepanocytes, and other abnormal forms, as well as leukocytes and platelets. The largest groups were hypochromic cells and spherocytes, each exceeding 20% of the dataset, while rare forms such as Howell–Jolly bodies (0.34%), Heinz bodies (0.01%), and drepanocytes (0.18%) were also identified. Figure 10 illustrates representative examples of validated morphologies, including target cells, teardrop cells, and schistocytes. The validation process produced a dataset containing clinically diverse red blood cell morphologies, providing reliable and well-structured samples suitable for analysis.

### 3.4. Data Augmentation

**Figure 11:**
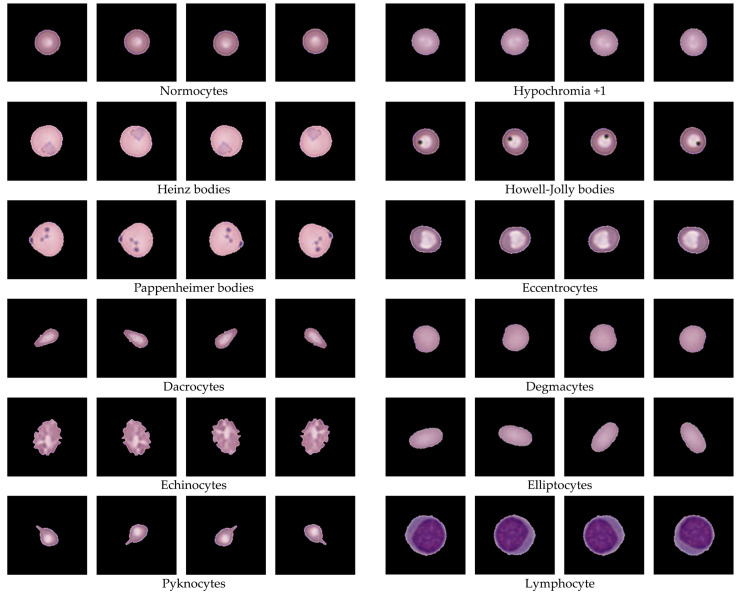
Representative examples of RBC data augmentation, showing generated images from scaling (S), rotation (R), and flipping (F). The images illustrate morphological preservation while increasing dataset diversity and demonstrate how augmentation produced realistic variations across both common and rare cell types.

Data augmentation was applied to expand the dataset and address class imbalance across morphological categories. Following this process, each class was systematically increased to either 1000 or 4000 images, depending on the target balancing requirements. In particular, rare morphologies such as Heinz bodies, Howell–Jolly bodies, Pappenheimer bodies, keratocytes, and drepanocytes, which were poorly represented in the original dataset, received the largest proportional increases. This ensured that classes with fewer than 50 original samples were sufficiently enlarged to contribute to downstream training. Representative examples of augmented images are presented in Figure 11. The generated cells demonstrate preserved morphology following scaling, rotation, and flipping operations, showing that augmentation enhanced data diversity while maintaining realistic visual characteristics of RBCs. A complete summary of augmentation is provided in Table 6, which lists the number of original inputs, the operations applied, and the final class sizes. After augmentation, the dataset exhibited a more balanced representation of both common and rare categories.

## 4. Discussion

Our study demonstrated several key findings through a hybrid framework integrating preprocessing, unsupervised feature learning, geometric analysis, and expert refinement. Firstly, the preprocessing pipeline systematically isolated over 34,000 high-quality single-cell images (Table 3 and Table 4) while filtering out artifacts, overlapping cells, and edge-cut regions, thereby providing clean inputs for downstream analysis and supporting quality control in hematology laboratories. Secondly, unsupervised clustering using autoencoder-derived latent features proved effective for grouping morphologically coherent RBCs. Figure 6 shows that CNN-based autoencoders achieved lower reconstruction losses compared with dense models, and Figure 7 demonstrates that clustering performance peaked at k = 80, with UMAP visualization in Figure 8 further confirming improved separation. This aligns with prior studies showing that low reconstruction loss reflects robust latent encoding [43,55]. While dense autoencoders converged faster, CNN-based models better preserved spatial detail, consistent with evidence that convolutional architectures enhance cellular image representation [56]. The selection of k = 80 was supported not only by clustering metrics but also by prior evidence that fine-grained clustering improves feature grouping in medical imaging [45,57], yielding interpretable clusters without excessive fragmentation. Thirdly, ellipse-based geometric priors improved discrimination of subtle traits such as elongation, circularity, and completeness, in accordance with hematological standards [52,53,54]. Representative results of this step are shown in Figure 9, while expert review further validated 36 morphologies summarized in Table 5 and illustrated in Figure 10. This step provided quantitative interpretability that strengthened the overall framework. Fourthly, expert-in-the-loop validation confirmed 36 clinically meaningful morphologies, ensuring that annotations retained diagnostic reliability while reducing expert workload. Finally, targeted data augmentation enriched rare morphological subtypes with biologically plausible variants, as summarized in Table 6 and illustrated in Figure 11, mitigating class imbalance that often limits sensitivity in hematology AI models.

Beyond technical performance, the dataset also carries important clinical implications that enhance its translational value. The predominance of hypochromic, codocytic, and spherocytic cells (Table 5) closely mirrors hematological profiles commonly observed in thalassemia syndromes and related anemias across Southeast Asia, particularly within populations where HbE and β-thalassemia are highly prevalent. Figure 10 further illustrates representative examples of these morphologies, showing characteristic features of target cells, teardrop cells, and schistocytes that are frequently encountered in clinical hematology practice. This concordance indicates that the dataset not only captures morphological variability but also reflects the true clinical distribution of disease-related phenotypes in the region. By aligning with population-specific hematological patterns, the dataset provides a realistic foundation for the development of diagnostic models that can directly benefit local healthcare systems. Such an alignment is especially important because widely used datasets derived from Western cohorts often fail to generalize to Southeast Asian populations, where the prevalence of hemoglobinopathies is substantially higher. In this context, the inclusion of thousands of hypochromic and spherocytic cells alongside rarer but clinically significant forms such as Howell–Jolly bodies and drepanocytes (Table 5) ensures that the dataset maintains both breadth and depth of morphological representation. Moreover, by combining unsupervised clustering, quantitative geometric analysis, and expert-in-the-loop refinement, the framework ensures that annotations remain interpretable, clinically trustworthy, and reproducible. This integration reduces subjectivity, supports scalability, and enhances reproducibility, thereby positioning the dataset as a practical and clinically relevant resource for developing AI-assisted diagnostic systems that are directly applicable to regional practice.

Nevertheless, several limitations should be acknowledged when interpreting the findings of this study. ROI selection, although guided by quantitative thresholds, would benefit from further standardization to improve consistency and to better capture the relationship between local cell density and disease-specific morphological patterns. In the present work, touching or aggregated cells were excluded from the analysis, even though such configurations may hold diagnostic relevance in hematology, particularly in evaluating anisopoikilocytosis and rouleaux formation. Rare morphologies also remained underrepresented or absent. For example, Table 5 shows that basophilic stippling (0.01%), Howell–Jolly bodies (0.34%), Heinz bodies (0.01%), and drepanocytes (0.18%) accounted for only a minimal fraction of the dataset, while several categories such as HbH inclusions or Cabot rings were not observed at all. These imbalances limit broader applicability beyond anemia and thalassemia. Although data augmentation partially addressed this issue by expanding rare classes to 1000 or 4000 images (Table 6), augmentation cannot fully substitute for genuine clinical diversity. Future work should therefore refine ROI selection protocols, develop robust algorithms to segment touching cells, and expand datasets to capture additional rare morphologies. Further studies should also explore population-specific distributions of RBC forms in Thai cohorts and assess the diagnostic value of underrepresented categories. Beyond dataset enrichment, advanced architectures such as transformer-based models could improve both accuracy and interpretability, while multi-institutional collaborations will be essential for building larger and regionally diverse datasets that more comprehensively represent hematological variability and strengthen the clinical relevance of computational frameworks.

Another important limitation of the present study is the absence of a healthy control group. Because the dataset was derived exclusively from blood smears of patients with anemia and thalassemia, all morphological classes summarized in Table 5 represent abnormal or disease-associated RBC phenotypes. Without normal samples, the framework cannot evaluate its ability to distinguish “normal versus abnormal” cells, which is fundamental for diagnostic applications. Likewise, the lack of a healthy control group prevents systematic benchmarking of morphological markers that differentiate thalassemia from other anemias such as iron deficiency, where microcytic and hypochromic cells are also common but arise in a different clinical setting. In practice, this limits the immediate diagnostic utility of the dataset, as no baseline reference distributions are available for comparison. Although the inclusion of healthy controls would enhance clinical relevance, this was beyond the scope of the current study, which focused on documenting abnormal morphologies associated with thalassemia in Thai patients. The primary aim was to establish a locally derived framework for semi-automated RBC labeling and dataset construction, providing a methodological contribution to support machine learning applications. Thus, the dataset should be regarded as a resource for algorithmic development and computational hematology research, rather than a diagnostic reference standard. While the framework demonstrates feasibility and scalability, the absence of normal samples remains a critical gap for clinical translation. Future work will therefore prioritize expanding the dataset to include healthy controls as well as patients with additional hematological disorders beyond thalassemia. This will enable benchmarking of model specificity by comparing normal, thalassemic, and non-thalassemic anemias. For example, distinguishing iron deficiency anemia from thalassemia will require robust morphological references for normocytes, microcytes, and hypochromic cells across both healthy and diseased states. Inclusion of healthy samples will also allow systematic evaluation of false positive and false negative rates in automated classification, ensuring broader applicability. Furthermore, incorporating multi-institutional cohorts will strengthen population diversity, providing a stronger foundation for developing clinically deployable AI-assisted hematology systems.

Another limitation is the absence of a healthy control group. As shown in Table 5, all morphological categories in the dataset represent abnormal or disease-associated RBCs, and no normal samples were included. Without such controls, the framework cannot directly assess the ability to distinguish “normal versus abnormal” RBCs or evaluate markers differentiating thalassemia from other anemias such as iron deficiency. Although including healthy controls would enhance clinical relevance, this was beyond the scope of the present study, which focused specifically on abnormal morphologies in thalassemia cases. The primary aim was to establish a locally derived framework for semi-automated RBC labeling and dataset construction to support future deep learning applications. Thus, the dataset should be regarded as a methodological contribution rather than a diagnostic reference. Future work will expand the dataset with healthy controls and additional disorders to benchmark specificity and strengthen clinical applicability.

Together, these strengths demonstrate that the proposed framework not only advances methodological rigor but also provides practical value for hematological research. By integrating preprocessing, unsupervised feature learning, geometric priors, expert validation, and targeted augmentation, the approach establishes a reliable pipeline for scalable RBC labeling. The dataset incorporates 34,282 valid single-cell patches (Table 3 and Table 4), clustering outcomes optimized at k = 80 (Figure 7), quantitative geometric characterization through ellipse fitting (Figure 9), and expert validation confirming 36 morphologies (Table 5; Figure 10). Targeted augmentation further balanced rare classes (Table 6; Figure 11). The resulting dataset captures real-world smear variability from Thai patients, ensuring that morphological diversity and artifact complexity are adequately represented. This makes the dataset an important regional complement to existing Western-centric resources, which are often smaller, more curated, and less representative of local disease patterns. Consequently, the framework serves as both a robust foundation for future AI model development and a practical resource to support hematology laboratories, particularly in regions where expert availability is limited and scalable diagnostic support is urgently needed.

## 5. Conclusions

This study demonstrated a hybrid framework for RBC labeling that integrates preprocessing, unsupervised feature learning, ellipse fitting, expert-in-the-loop validation, and targeted augmentation. The framework successfully generated a dataset of over 14,000 high-quality single-cell images from Thai patient smears, organized into 36 clinically meaningful morphologies. Clustering at k = 80 provided interpretable groups supported by both quantitative metrics and expert review, while ellipse-based geometric analysis improved discrimination of subtle shape variations. Expert validation further ensured diagnostic reliability, and augmentation enhanced representation of rare classes. Together, these steps establish a scalable and interpretable methodology for RBC annotation that bridges automated clustering with clinical expertise. Although not yet designed for diagnostic discrimination, the framework provides a solid foundation for future work in expanding datasets, incorporating healthy controls, and training advanced deep learning models for hematological research.

## Figures and Tables

**Table 1:** A concise comparison of representative methods in RBC image analysis, highlighting their main advantages and limitations.

Method	Advantages	Limitations	References
WSI and ROI Extraction	High-resolution slides; full context	Lacks adaptive ROI; poor standardization	[16,17]
Single-Cell Segmentation	Efficient (classic); accurate (DL)	Fails on overlaps; needs dense labels	[18,19]
Unsupervised Clustering	Reduces labeling cost; latent features	Few RBC studies; limited backbone comparison	[20,21]
Shape-Based Modeling	Simple; interpretable; ellipse captures geometry	Weak on noisy smears; rarely combined with DL	[22,23,24,25]
HITL Refinement	Less expert workload; higher trust	Rarely used in RBC; weak integration tools	[26,27,28,29]

**Table 2:** Six WSIs from Thai patients with confirmed hematological diagnoses were analyzed. Cases span from anemia to thalassemia, from mild iron deficiency to severe homozygous Hb E. Severity followed clinical guidelines, ensuring representation of both common and rare RBC morphological subtypes.

Sample ID	Diagnosis	Abbreviation	Condition Severity
01	Iron Deficiency Anemia	IDA	Mild to moderate
02	Thalassemia Trait	TT	Carrier (asymptomatic)
03	Hb H Disease	HbH	Moderate to severe
04	Hb E/β-thalassemia	HbE/β-thal	Variable (mild–moderate)
05	Hb E/β-thalassemia with severe symptoms	HbE/β-thal Sx	Severe
06	Homozygous Hb E Thalassemia	Homo HbE	Severe

**Table 3:** Distribution of Dataset 1 cell categories across six hematological conditions. Only isolated single-cell patches were retained for classification, while other categories show excluded cells during preprocessing.

Sample	Single Cells	Extracted Cells	Overlapping	Small Cells	Touching Edge	Other
IDA	733	132	20	168	25	0
TT	1124	65	17	94	50	0
HbH	1551	379	427	328	70	0
HbE/β-thal	5009	732	476	590	104	0
HbE/β-thal Sx	930	445	211	853	63	0
Homo HbE	2803	148	93	204	68	0
Total	12,150	1901	1244	2237	380	0

**Table 4:** Distribution of Dataset 2 categories, including clusters, overlapping, fragmented, debris, and truncated cells. These were excluded from model training but retained for quality control and artifact documentation.

Sample	Single Cells	Extracted Cells	Overlapping	Small Cells	Touching Edge	Other
IDA	785	164	30	853	35	0
TT	1874	239	381	298	59	0
HbH	1167	270	362	232	68	0
HbE/β-thal	2443	640	496	415	71	0
HbE/β-thal Sx	381	280	174	723	49	0
Homo HbE	3013	240	271	291	66	0
Total	9663	1833	1714	2812	348	0

**Table 5:** Distribution of 14,089 single-cell RBC images after expert validation, summarized across staining alterations, inclusions, hemoglobin distribution, shape variations, leukocytes, platelets, and other categories. Counts and percentages indicate the relative frequency of each morphological class within the dataset.

Class Name	Morphological Name	Count	Percentage
Normocytes	Normocytes *	805	5.75%
Alteration in staining	Hypochromia + 1 *	1698	12.13%
	Hypochromia + 2 *	1059	7.56%
	Hypochromia + 3 *	240	1.71%
	Hypochromia + 4 *	47	0.34%
Erythrocyte inclusions	Basophilic stippling	1	0.01%
	HbH inclusions	0	0.00%
	Diffuse basophilia	0	0.00%
	Cabot ring	0	0.00%
	Hb H	0	0.00%
	Hb C crystal	0	0.00%
	Hb SC crystal	0	0.00%
	Heinz bodies	2	0.01%
	Howell-Jolly bodies	47	0.34%
	Pappenheimer bodies	16	0.11%
Variations in Hb distribution	Codocytes-01 *	1024	7.31%
	Codocytes-02 *	1050	7.50%
	Eccentrocytes	202	1.44%
	Spherocytes-01 *	1718	12.27%
	Spherocytes-02 *	1205	8.61%
	Stomatocytes	173	1.24%
Variations in RBCs shape	Acanthocytes	16	0.11%
	Dacrocytes	396	2.83%
	Degmacytes	393	2.81%
	Drepanocytes	25	0.18%
	Echinocytes	27	0.19%
	Elliptocytes *	136	0.97%
	Keratocytes	7	0.05%
	Knizocytes	525	3.75%
	Ovalocytes *	0	0.00%
	Pyknocytes	603	4.31%
	Schistocytes	488	3.49%
Leukocytes	Basophil	1	0.01%
	Eosinophil	0	0.00%
	Lymphocyte	21	0.15%
	Monocyte	2	0.01%
	Neutrophil	9	0.06%
Platelets	Platelets-01 *	312	2.23%
	Platelets-02 *	61	0.44%
Others	Large-01 *	766	5.47%
	Large-02 *	537	3.84%
	Small	117	0.84%
	Other	271	1.94%
Total		14,089	100.00%

* Ellipse fitting was applied to classify abnormal RBC shapes, refining geometric boundaries and improving accuracy in morphological analysis.

**Table 6:** Summary of data augmentation across morphological classes. The table reports original input counts, the specific augmentation operations applied, and the final target sizes of either 1000 or 4000 images per class, demonstrating improved balance between frequent and rare categories within the dataset.

Label List	Input	Augmentation	
		1000 Images	4000 Images
Normocytes	50	R (5), F (3)	R (20), F (3)
Hypochromia + 1	50	R (5), F (3)	R (20), F (3)
Hypochromia + 2	50	R (5), F (3)	R (20), F (3)
Hypochromia + 3	50	R (5), F (3)	R (20), F (3)
Hypochromia + 4	25	R (10), F (3)	R (40), F (3)
Basophilic stippling	0	-	-
HbH inclusions	0	-	-
Diffuse basophilia	0	-	-
Cabot ring	0	-	-
Hb H	0	-	-
Hb C crystal	0	-	-
Hb SC crystal	0	-	-
Heinz bodies	2	R (125), F (3)	S (2), R (250), F (3)
Howell-Jolly bodies	25	R (10), F (3)	R (40), F (3)
Pappenheimer bodies	10	R (25), F (3)	R (100), F (3)
Codocytes-01	250	R (1), F (3)	R (4), F (3)
Codocytes-02	250	R (1), F (3)	R (4), F (3)
Eccentrocytes	125	R (2), F (3)	R (4), F (3)
Spherocytes-01	250	R (1), F (3)	R (4), F (3)
Spherocytes-02	250	R (1), F (3)	R (4), F (3)
Stomatocytes	50	R (5), F (3)	R (20), F (3)
Acanthocytes	10	R (25), F (3)	R (100), F (3)
Dacrocytes	50	R (5), F (3)	R (20), F (3)
Degmacytes	25	R (10), F (3)	R (40), F (3)
Drepanocytes	25	R (10), F (3)	R (40), F (3)
Echinocytes	25	R (10), F (3)	R (40), F (3)
Elliptocytes	50	R (5), F (3)	R (20), F (3)
Keratocytes	5	R (50), F (3)	R (200), F (3)
Knizocytes	125	R (2), F (3)	R (4), F (3)
Ovalocytes	125	R (2), F (3)	R (4), F (3)
Pyknocytes	125	R (2), F (3)	R (4), F (3)
Schistocytes	125	R (2), F (3)	R (4), F (3)
Basophil	0	-	-
Eosinophil	0	-	-
Lymphocyte	25	R (10), F (3)	R (40), F (3)
Monocyte	2	R (125), F (3)	S (2), R (250), F (3)
Neutrophil	10	R (25), F (3)	R (100), F (3)
Platelets-01	50	R (5), F (3)	R (20), F (3)
Platelets-02	50	R (5), F (3)	R (20), F (3)
Large-01	250	R (1), F (3)	R (4), F (3)
Large-02	250	R (1), F (3)	R (4), F (3)
Small	50	R (5), F (3)	R (20), F (3)
Other	250	R (1), F (3)	R (4), F (3)

**Figure A1:**
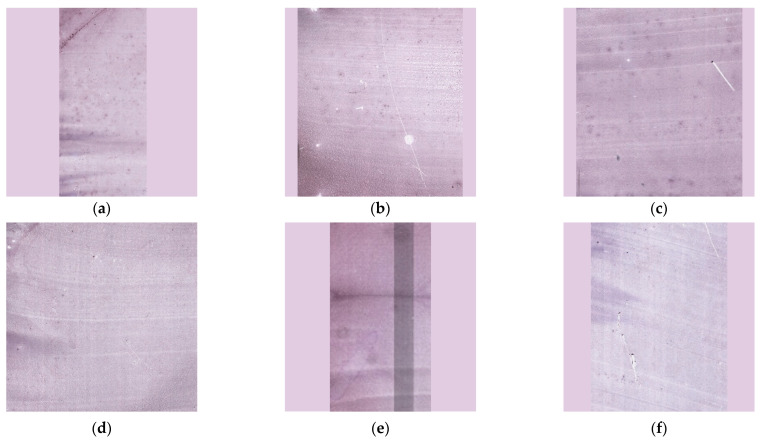
Thumbnails of hematology samples derived from WSI different types of thalassemia, including (**a**) IDA, (**b**) TT, (**c**) HbH, (**d**) HbE/β-thal, (**e**) HbE/β-thal Sx, and (**f**) Homo HbE.

**Figure A2:**
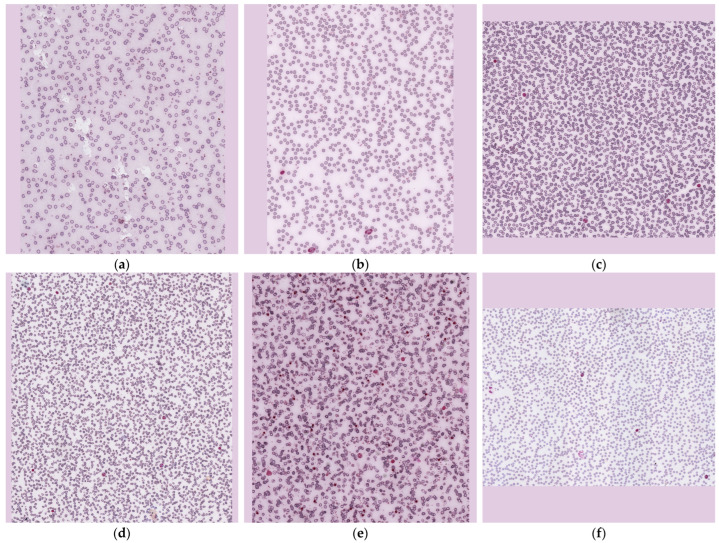
Representative examples of ROI extractions from each sample WSI, selected based on diagnostic relevance and cell distribution, including (**a**) IDA, (**b**) TT, (**c**) HbH, (**d**) HbE/β-thal, (**e**) HbE/β-thal Sx, and (**f**) Homo HbE.

## Data Availability

The data supporting the reported results of this study are not publicly available due to privacy and ethical restrictions related to patient confidentiality. However, de-identified datasets may be made available from the corresponding author upon reasonable request and with appropriate ethical approval.

## Abbreviations

The following abbreviations are used in this manuscript:

AI	Artificial Intelligence
AR	Aspect Ratio
CNN	Convolutional Neural Network
ER	Ellipse-to-cell Area Ratio
GAN	Generative Adversarial Network
HbE	Thalassemia Hb E Disease
HbE Sx	Thalassemia Hb E Disease with Severe Symptoms
HbH	Hemoglobin H Disease
Ho HbE	Homozygous Hb E Thalassemia
HITL	Human-in-the-Loop
ID	Identifier
IDA	Iron Deficiency Anemia
OpenCV	Open Source Computer Vision Library
PLT	Platelet
RBC	Red Blood Cell
ReLU	Rectified Linear Unit
ROI	Region of Interest
SVS	Scanned Virtual Slide Format
TT	Thalassemia Trait
UMAP	Uniform Manifold Approximation and Projection
U-Net	U-shaped Convolutional Neural Network
WBC	White Blood Cell
WSI	Whole Slide Image

## Appendix A

### Appendix A.1

The study analyzed six WSIs in SVS format representing various hematological conditions: IDA, TT, HbH, HbE/β-thal, HbE/β-thal Sx, and Homo HbE. Each WSI was processed using Python with the OpenSlide library to extract essential metadata, including pixel size (0.1658 µm), magnification power (83×), number of image levels (4), and dimensions at the highest resolution. This metadata is critical for validating image quality and ensuring compatibility for downstream image processing tasks. In addition, thumbnails were generated and displayed for visual inspection, allowing verification of staining quality, smear uniformity, and potential artifacts, as illustrated in Figure A1. These thumbnails facilitated rapid screening prior to computational analysis, reducing the likelihood of processing flawed images. The extracted metadata and quality assessment outcomes are summarized in Table A1, which confirms consistency across all samples. This step ensured standardized, high-resolution inputs for subsequent preprocessing and provided a reliable baseline for comparing image characteristics across disease-specific samples. Moreover, this process demonstrates an effective workflow for digitizing and validating hematological slides, offering reproducible methodology for dataset preparation and serving as a reference for future large-scale RBC morphology studies.

**Table A1:** The extracted properties of pathology scanning slide.

Sample Name	Pixel (µm)	Magnification	Levels	Dimensions (Pixels)
IDA	0.1658	83	4	34,271 × 74,047
TT	0.1658	83	4	44,743 × 51,260
HbH	0.1658	83	4	46,647 × 52,973
HbE/β-thal	0.1658	83	4	52,359 × 51,740
HbE/β-thal Sx	0.1658	83	4	39,031 × 73,061
Homo HbE	0.1658	83	4	39,983 × 55,429

### Appendix A.2

Appendix A.2 illustrates examples of ROIs extracted from the six hematological samples, as shown in Figure A2, highlighting the representative areas selected for analysis based on cell density and image quality. Additionally, Table A2 summarizes the dimensions of the two ROIs extracted per sample, which were used to ensure coverage of diagnostically relevant areas while maintaining variability across datasets. This systematic ROI selection provided standardized inputs for subsequent preprocessing and feature extraction steps.

**Table A2:** Dimensions of ROI 1 and ROI 2 (in pixels) for each sample analyzed in this study.

Sample	Dimensions (Pixels)	
	ROI 1	ROI 2
IDA	2358 × 2882	2489 × 2751
TT	2489 × 3340	4575 × 3275
HbH	4519 × 3733	3733 × 3471
HbE/β-thal	7991 × 4454	4454 × 5043
HbE/β-thal Sx	3144 × 5305	3013 × 3471
Homo HbE	5305 × 4061	5436 × 3995
